# The abundance and diversity of arbuscular mycorrhizal fungi are linked to the soil chemistry of screes and to slope in the Alpic paleo-endemic *Berardia subacaulis*

**DOI:** 10.1371/journal.pone.0171866

**Published:** 2017-02-13

**Authors:** Gabriele Casazza, Erica Lumini, Enrico Ercole, Francesco Dovana, Maria Guerrina, Annamaria Arnulfo, Luigi Minuto, Anna Fusconi, Marco Mucciarelli

**Affiliations:** 1 Università di Genova, DISTAV, Corso Europa 26, GENOVA, Italy; 2 Istituto per la Protezione Sostenibile delle Piante–CNR, Viale P.A. Mattioli 25, TORINO, Italy; 3 Dipartimento di Scienze della Vita e Biologia dei Sistemi, Università di Torino, Viale P.A. Mattioli 25, TORINO, Italy; Estacion Experimental del Zaidin, SPAIN

## Abstract

*Berardia subacaulis* Vill. is a monospecific genus that is endemic to the South-western Alps, where it grows on alpine screes, which are extreme habitats characterized by soil disturbance and limiting growth conditions. Root colonization by arbuscular mycorrhizal fungi (AMF) is presumably of great importance in these environments, because of its positive effect on plant nutrition and stress tolerance, as well as on structuring the soil. However, there is currently a lack of information on this topic. In this paper, we tested which soil characteristics and biotic factors could contribute to determining the abundance and community composition of AMF in the roots of *B*. *subacaulis*, which had previously been found to be mycorrhizal. For such a reason, the influence of soil properties and environmental factors on AMF abundance and community composition in the roots of *B*. *subacaulis*, sampled on three different scree slopes, were analysed through microscopic and molecular analysis. The results have shown that the AMF community of *Berardia* roots was dominated by Glomeraceae, and included a core of AMF taxa, common to all three scree slopes. The vegetation coverage and dark septate endophytes were not related to the AMF colonization percentage and plant community did not influence the root AMF composition. The abundance of AMF in the roots was related to some chemical (available extractable calcium and potassium) and physical (cation exchange capacity, electrical conductivity and field capacity) properties of the soil, thus suggesting an effect of AMF on improving the soil quality. The non-metric multidimensional scaling (NMDS) ordination of the AMF community composition showed that the diversity of AMF in the various sites was influenced not only by the soil quality, but also by the slope. Therefore, the slope-induced physical disturbance of alpine screes may contribute to the selection of disturbance-tolerant AMF taxa, which in turn may lead to different plant-fungus assemblages.

## Introduction

A great deal of literature exists on the global species richness and distribution of mycorrhizal and non-mycorrhizal endophytic fungi ([1–3]). Arbuscular mycorrhizal fungi (AMF), or Glomeromycota [4], are obligate symbiotic fungi that penetrate plant roots and form the arbuscule, that is, a specialized hyphal structure that develops inside cortex cells, and represents the main site of nutrient exchange between partners [5]. These fungi have played an important role in the evolution of land plants for more than four hundred million years [6], and they today colonize the roots of most plants [5]. In turn, the plants, despite their ability to live independently, may increase nutrient uptake, growth and reproductive success when associated with AMF [5]. Moreover, AMF ameliorate soil quality [5] and improve the ability of host plants to withstand abiotic stress and disease [7], thus increasing plant performances [8]. The host plants of AMF are usually co-colonized by non-mycorrhizal endophytic fungi, including dark septate endophytes (DSEs). The latter are characterized by melanised, septate hyphae that either extracellularly or intracellularly colonize plant roots ([9],[10]). DSEs have been shown to influence plant growth and physiology [11], even though the role of these endophytes in plant fitness is less clear than for mycorrhizal fungi [10].

The intensity and diversity of AMF colonization have been shown to be low at high altitudes in different mountain environments ([12–18]), although contrasting results have been reported (see [12],[17]), particularly for the Alps ([19]-[23]). AMF diversity is generally influenced by the soil chemistry, and especially by the soil pH [24], while the effects of other factors such as plant community [25] and soil disturbance [26] are less clear.

*B*. *subacaulis* Vill. (Asteraceae) is a rare species that is endemic to the South-western Alps, which grows exclusively on high-altitude, calcareous screes (1,700–2,700 m asl). Preliminary surveys have shown that this species is colonized by AM and DSE fungi (Mucciarelli and Fusconi, unpublished). This plant plays an important role in the conservation of alpine biodiversity, because it belongs to a monospecific genus, and represents an old Tertiary lineage [27], with no close extant relatives [28]. The plant is believed to have survived the climate changes of the past, and to have persisted in extreme habitats with low inter-specific competition and few pollinators because of the lack of floral specialization and the possibility of self-pollination ([29],[30]). However, a great reduction in habitat suitability has been predicted for *Berardia* under future climate change scenarios [30], on the basis of the results of species distribution modelling.

In the last few years, a great effort has been made to understand the factors that shape the AMF community throughout the world. This paper is an attempt to add information to this body of knowledge analysing the determinants of the abundance and community composition of AMF in the roots of *B*. *subacaulis* Vill. To achieve this goal, plants from three alpine sites in the South-West of Piedmont (Italy) and in the nearby part of France were sampled, and their percentages of root colonization and AMF diversity were determined.

Moreover, because the *B*. *subacaulis* habitat is represented by sloping alpine screes, characterized by a recurring downward movement of rock fragments and soil, the resulting data were considered in relation to the morphological characteristics of soil, including the slope, and to some physical-chemical properties of the soil, focusing on those linked to soil structure and stability. Since biotic factors are known to influence AMF populations, plant species abundance, vegetation coverage and colonization by DSE in the roots of *B*. *subacaulis* were also considered.

## Materials and methods

### Study sites and sample collection

During the summer of 2014, three sites were chosen to represent the typical habitat of *B*. *subacaulis* (Table 1). These sites are characterized by unstable calcareous screes, consisting of coarse free-draining debris and sparse patches of basic loam. Because the availability of phosphate and the pH are known to influence the abundance and community of AMF ([5],[24]), three sites with only slight differences in these parameters were chosen.

**Table 1:** Details of the sampling sites.

Site	Code	Longitude	Latitude	Altitude (m asl)
Bassa di Colombart (IT)	CLM	6.91673	44.36068	2345
Millefonts (FR)	MIL	7.18642	44.09843	2039
Valcavera (IT)	VAL	7.09784	44.38426	2408

From four to five non-neighbouring plots (5m × 5m) were randomly selected (N = 14) at each site. Because of the rarity of this species, only one plant was dug up from each plot for the subsequent microscopic and molecular investigations. Specific permission for plant sampling at Bassa di Colombart (CLM, Argentera, CN, Italy) and Valcavera (VAL, Demonte, CN, Italy) was not required since the two studied sites are outside protected areas and *Berardia subacaulis* is not an endangered nor a protected species in Italy (according to Art. 15 of the Regional Law 82/32 which regulates the number of samples of the plant species for which collection in the wild is allowed, Piedmont, Italy). A specific permission to collect five plants at the site of Millefonts (Valdeblore, France) was issued by the Conservatoire Botanique National Méditerranéen de Porquerolles (CBNMED, 34 Avenue Gambetta, 83400 Hyères, France) within a collaborative research project between the CBNMED and the University of Genova (Italy) aimed at improving the knowledge of the biology of the species. Selected individuals were at full bloom and similar in size, with 3–4 fully developed leaves (S1 Fig). The main morphological characteristics of the soils (slope, bare soil and stone coverage), vegetation coverage and phytosociological relevés were recorded in each plot. A soil sample was collected from each plot at a depth of 20–40 cm (in at least three different places in each plot), and stored in a plastic bag, for later determination of the physical-chemical properties of the soil, that is, the soil properties. The air-dried samples were sieved through a 0.20 mm mesh before the total nitrogen, total carbonate, active carbonate, available extractable nutrients (P, K, Mg, Na, Ca), pH, carbon/nitrogen ratio, organic matter, cation exchange capacity (CEC), electrical conductivity (EC) and field capacity (FC) were measured. The properties and characteristics of the soil and vegetation coverage are reported in S1 Table. The analyses were performed by the Regione Liguria—Servizi alle Imprese Agricole e Florovivaismo, Laboratorio Regionale Analisi Terreni e Produzioni Vegetali (Sarzana, Italy). Lateral roots of *B*. *subacaulis* root samples were detached from the tap root at the same depth as that considered for the soil analysis in order to conduct microscopic and molecular analyses.

### Percentage of root colonization

The roots of different thickness of each plant were cut into segments of about 5 mm and randomly pooled into three samples. They were then cleared and stained with trypan blue, according to the usual procedures [31]. The percentages of total AM root colonization (AMF), arbuscules, AM vesicles, dark septate endophytes (DSE), and microsclerotia were estimated microscopically at a 200x magnification, by means of the line-intercept method [32], as the percentage of the fungal structure found to the total of interceptions (about 300 interceptions per sample).

### Genomic DNA extraction, PCR amplification, cloning and sequencing

Two independent DNA extractions (0.5 g of fresh weight) were conducted on the 14 *B*. *subacaulis* root samples using a DNeasy Plant Mini Kit (Qiagen, Crawley, UK). The DNA extracts were stored at −20°C. Partial small subunit (SSU) ribosomal RNA gene fragments were amplified using nested PCR [33], with the universal eukaryotic primers NS1 and NS4 [34], and a subsequent amplification round with the Glomeromycota-specific primers AML1 and AML2 [35]. Although longer and higher discriminating regions are available [36], the AML1/AML2 SSU region was targeted because most Glomeromycota diversity data are obtained using this region, which provides a larger comparative DNA sequence data-set. PCR was carried out using 0.2 mM dNTPs, 3.5 mM MgCl_2_, 0.5 μM of each primer, 2 units of GoTaq® (Promega, Milan, Italy), and the supplied reaction buffer, to obtain a final volume of 20 μl. Amplifications were carried out in 0.2 ml PCR tubes using a Biometra T Gradient thermocycler, according to the following steps: 5 min initial denaturation at 94°C, 35 cycles of 1 min at 94°C, 1 min at 55°C and 58°C for the two nested PCR rounds, respectively; 1 min at 72°C; and a final elongation of 10 min at 72°C. All the PCR products were checked using 1.5% agarose gel stained with ethidium bromide (Sigma-Aldrich, Milan, Italy). The four nested PCR product replicates were pooled and purified using Wizard® SV Gel and a PCR Clean-Up System kit (Promega). Before ligation, the quantity and quality of the PCR amplicons were checked using a spectrophotometer (NanoDrop Technology, Wilmington, DE). Cloning was done using the pGEM-T vector system (Promega), and transformed into *Escherichia coli* (Xl1 blue). At least 40 recombinant clones per amplicon library (No. = 14) were screened for the AML1/AML2 fragment (ca. 800 bp) on agarose gels. The clones were sequenced, using either the universal primer SP6 or T7, by LMU sequencing services (Munich, Germany).

### Sequence analyses and phylogenetic inference

Sequence editing was done using Sequencher V4.2.2 (Gene Codes Corporation, Ann Arbor, MI, USA). Potential chimera sequences were identified using the Chimera UCHIME algorithm implemented in Mothur v1.33.3 for Mac [37]. All the sequences were aligned using the multiple sequence comparison alignment tool in MAFFT v6 [38]. Distance matrices were constructed using the *dist*.*seqs* function implemented in Mothur. These pairwise distances were used as input to cluster the sequences into Operational Taxonomic Units (OTUs) of a defined sequence identity. A threshold of 97% identity was used to define the OTUs. Although this distance cut-off is arbitrary, and can be considered controversial, it was chosen on the basis of previous studies on AMF biodiversity ([33],[39]). A search for similar sequences was conducted with Blast v2.2.29 [40] using the latest release of the MaarjAM AMF Virtual Taxa database (classified as VTxy, where “xy” is a numerical code) [41], integrated with the SSU Silva database [42], cleared of Glomeromycota sequences. The results of two major reorganizations of the Glomeromycota classification have recently been published ([4],[21]). In this study, for ease of data handling, the phylogenesis derived from the work of Schüßler and Walker ([43],[44]) was adopted to affiliate the OTUs to the corresponding taxonomy. Since the ribosomal DNA fragment under study can make it difficult to phylogenetically separate some of the genera described in [43], clades were sometimes used (i.e. *Rhizophagus*/*Sclerocystis*, *Funneliformis*/*Septoglomus*, and *Glomus* sensu lato) in order to group sequences with a conservative approach. Any Non-Glomeromycota OTUs were removed from the dataset.

Phylogenetic analysis was performed on the sequences obtained in the present study, and on representative sequences retrieved from the MaarjAM AMF Virtual Taxa database [41]. Phylogenetic analyses were performed using MEGA6 [45]. MUSCLE implemented in MEGA6 was used as an alignment algorithm (default parameters). Neighbour-joining (NJ), with 1000 bootstrap replicates, and a Kimura 2-parameter model were used as the tree-building method. *Corallochytrium limacisporum* (L42528) was used as the outgroup taxon.

### Statistical analyses

Four different matrices were generated to perform the statistical analyses. Firstly, an abundance matrix was compiled in which the number of different OTU sequences in a given plot was reported. In order to ensure that the abundance of dominant and rare phylotypes contributed equally to the resultant matrix, the data were Hellinger-transformed [46]. A second matrix was compiled in which the percentage of root colonization was reported. A third environmental matrix was compiled in which the soil properties, the soil morphological features (slope, stone coverage and bare soil) and the vegetation coverage were reported. Percentage of plant coverage was estimated using the phytosociological relevés matrix. Finally, a fourth plant species abundance matrix was compiled transforming the Braun Blanquet scale used in phytosociological relevés to cover percentage using ‘simba’ R package [47] and data was Hellinger-transformed [46].

A species accumulation curve was generated, using the Ugland et al. method [48], to examine whether the number of OTUs increases as the sample size increases. The following were calculated for each plot in order to assess the differences in AMF community structure: taxon richness, Shannon diversity [49], Simpson’s dominance [50] and Fisher’s alpha, which is robust for comparisons among samples of different sizes [51]. A Kruskal-Wallis test was run to test for the overall differences in the diversity indices and percentage of colonization between the sampling sites, and a non-parametric Nemenyi–Damico–Wolfe–Dunn post hoc test was used to detect the pairwise differences between sites. An indicator species analysis was carried out using the ‘multipatt’ function in the ‘indicspecies’ R package, with 999 permutations [52], in order to assess whether the fungi were significantly associated with a particular locality. This is a classification-based method that is used to measure associations between species and groups of sampling sites [53]. Significance was calculated using 10,000 random interactions, and the significance level was set at P < 0.05. The distinctiveness of vegetation relevés on each locality was tested with an analysis of similarities (ANOSIM) in R using 10,000 permutations. Furthermore, the difference in plant species abundance between the plots of the different sites was assessed using the non-parametric Nemenyi–Damico–Wolfe–Dunn post hoc test. Indicator species analysis was carried out in order to assess whether the plant species were significantly associated with each locality.

In order to elucidate whether the environmental variables influenced the endophyte fungal communities, a non-metric multidimensional scaling (NMDS) was run with the ‘metaMDS’ function, and Bray–Curtis dissimilarities among plots calculated with multiple restarts (no. = 1,000) using ‘vegan’ R package [54]. The ‘envfit’ function in ‘vegan’ was used to determine the relationships between the AMF composition and the soil properties and characteristics. A Kendall tau correlation coefficient was employed to determine the relationships between the percentage of AMF colonization and the soil properties, the soil morphology, the vegetation coverage and the DSE colonization.

## Results

### AM root colonization and fungal diversity of *B*. *subacaulis*

**Fig 1:**
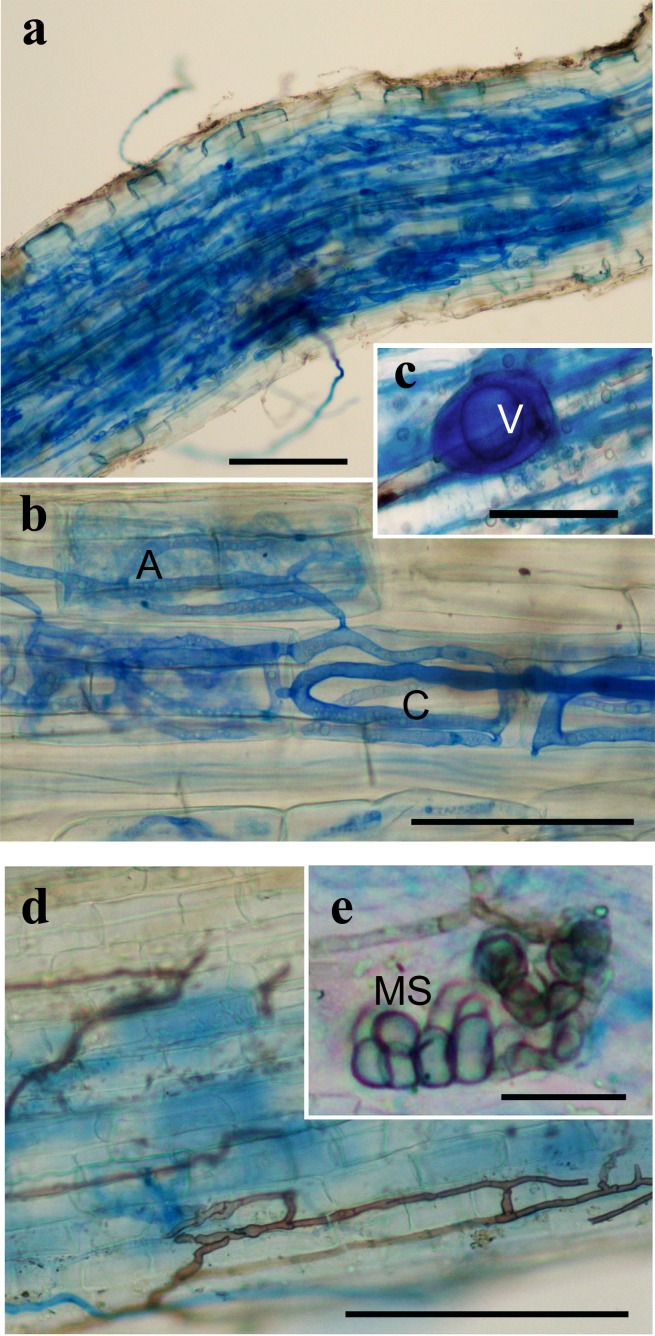
**Representative images of *B*. *subacaulis* roots colonized by arbuscular mycorrhizal fungi (AMF, a-c) and dark septate endophytes (DSE, d-e)**. (a) Extensive AMF colonization; (b) arbuscules (A) and intracellular hyphal coils (C); (c) intercellular vesicle (V); (d) DSE hyphae growing on the root epidermis and inside the cortex; (e) microsclerotium (MS); a, bar = 500 μm; b-d, bar = 100 μm; e, bar = 20 μm.

The AM colonization of the *B*. *subacaulis* roots ranged between about 37 and 60% of the root length, with no significant differences in percentage of colonization between the three locations. Arbuscules occurred along 61 to 85% of the colonized root lengths, while the occurrence of vesicles was lower. The total colonization of DSE was significantly higher in Bassa di Colombart (CLM) than in Millefonts (MIL), while the microsclerotia percentage was higher in CLM with respect to Millefonts (MIL) and to Valcavera (VAL) (Table 2 and Fig 1).

**Table 2:** Percentage of Fungal Colonization of the *B*. *subacaulis* Roots. The mean values ± SE of five root apparatus replicates are given as percentages of the root length.

Sampling sites	AMF			DSE	
	Total	AC	VC	Total	MS
CLM	44.23 ± 5.98*a*	37.53 ± 5.67*a*	20.37 ± 4.83*a*	33.38 ± 2.80*a*	12.87 ± 2.85*a*
MIL	37.30 ± 9.11*a*	24.39 ± 6.46*a*	15.93 ± 4.70*a*	15.73 ± 3.29*b*	1.86 ± 0.56*b*
VAL	59.91 ± 4.45*a*	36.91 ± 2.27*a*	20.53 ± 4.47*a*	17.51 ± 5.02*ab*	2.94 ± 1.82*b*

AMF, Arbuscular mycorrhizal fungi; AC, arbuscular colonization; VC, vesicular colonization; DSE, dark septate endophytes; MS, microsclerotia. CLM, Bassa di Colombart; MIL, Millefonts and VAL, Valcavera.

Values with the same letters do not differ significantly at P < 0.05 (Kruskal–Wallis test; post-hoc non-parametric Nemenyi–Damico–Wolfe–Dunn test).

**Fig 2:**
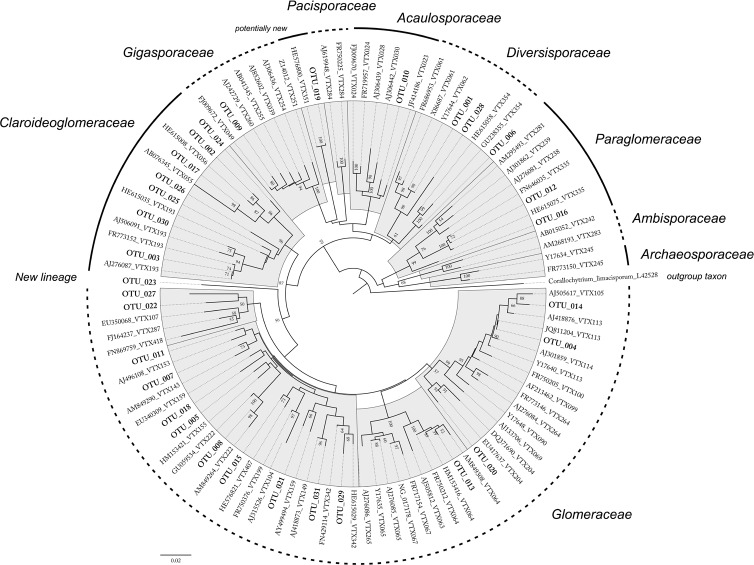
Phylogenetic tree showing the placement of the AM fungal OTUs associated with *B*. *subacaulis*. Reference sequences were retrieved from the MaarjAM AMF Virtual Taxa database [41]. *Corallochytrium limacisporum* (L42528) was used as the outgroup taxon.

Template DNA from 14 root samples of *B*. *subacaulis* was successfully amplified with the AML1/AML2 primer combination, and PCR products of the expected size (ca. 800 bp), which were then used to create clone libraries, were obtained. Overall, 560 clones were screened by means of PCR; out of these, 510 contained the SSU rRNA gene fragment. After preliminary BLASTn searches, a total of 380 clones were found to correspond to the AMF sequences, while the remaining clones were mainly identified as plant sequences (22.4%, data not shown). The AMF sequences were grouped into 31 OTUs, and the correspondence between the OTUs and the closest VT, after a blast search in the MaarjAM database, is shown in S2 Table. The 31 representative OTU sequences were registered in GenBank, under the following accession number: KY416573-KY416603, and are shown in bold in the phylogenetic tree in Fig 2.

**Fig 3:**
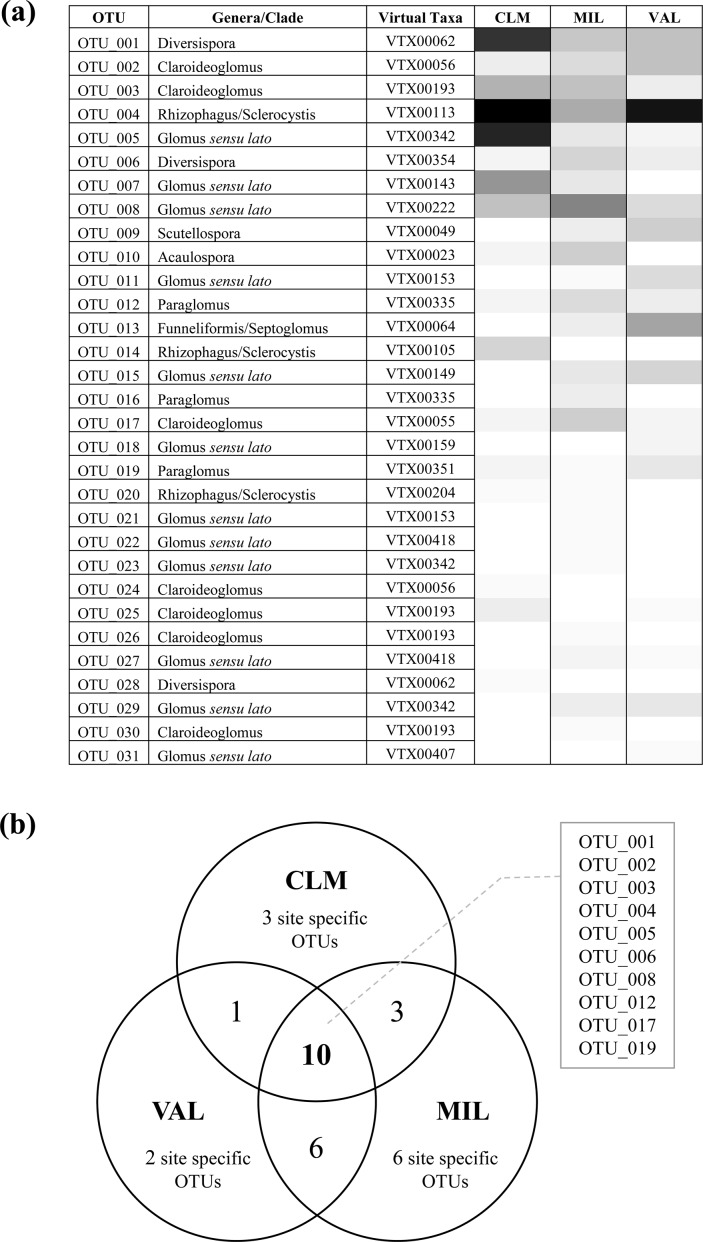
(a) **The ordinated heat-map based on occurrence classes (<1% absent, white, >30% dominant, black; light to dark grey, intermediate percentages) of the fungal operational taxonomic units (OTU’s, rows).** The genera, or clade, and the virtual taxa assignments are also indicated for the 31 OTUs. (b) **Venn diagram showing the number of shared and site-specific AMF OTUs. (Bassa di Colombart, CLM; Millefonts, MIL; Valcavera, VAL).**

Three out of four orders of the phylum Glomeromycota [4] were retrieved, thus indicating a good coverage of the biodiversity by the used primers [55]. The sequences were distributed over six families (Glomeraceae, Claroideoglomeraceae, Paraglomeraceae, Diversisporaceae, Gigasporaceae, and Acaulosporaceae) (Fig 2) or eight clades/genera (see S2 Table). The most abundant and diverse group in the roots of the *B*. *subacaulis* samples was, by large, the Glomeraceae, which represented 52% of the total sequences grouped in 16 OTUs, and this was followed by the Claroideoglomeraceae (22%), represented by 7 OTUs, and by both the Diversisporaceae and Paraglomeraceae, which were represented by 3 OTUs each. The Acaulosporaceae and Gigasporaceae were both represented by one OTU each (S2 Table). Considering their position in the phylogenetic tree (Fig 2), the most abundant and diverse genus grouping were those ascribed to *Glomus* sensu lato (32%, 12 OTUs), followed by *Rhizophagus*/*Sclerocystis* (21%, 3 OTUs), *Claroideoglomus* (17%, 7 OTUs), *Diversispora* (10%, 3 OTUs), *Funneliformis*/*Septoglomus* (4%, 1 OTU), and *Paraglomus* (5%, 3 OTUs). The latter genus included OTU019, and probably represents a new clade, as was reported in Öpik et al. [44]. *Acaulospora* and *Scutellospora* were both represented less (3%, 1 OTU each) (Figs 2 and 3A). The sampling effort curve indicated that the number of analysed root samples was sufficient to provide coverage of the AMF diversity in *B*. *subacaulis*, since the curve almost reached the plateau (S2 Fig).

### AMF variations between the sampling sites

As shown in the Venn diagram (Fig 3B), the three sites shared 10 OTUs, out of which 3 (OTU004, OTU005 and OTU008) belonged to Glomeraceae (the first one to *Rhizophagus*/*Sclerocystis*, VTX00113, and the remaining two to *Glomus sensu lato*, VTX00342 and VTX00222), 3 (OTU002, OTU003, OTU017) to Claroideoglomeraceae (*Claroideoglomus*, VTX00056, VTX00194 and VTX00055), 2 (OTU001 and OTU006) to Diversisporaceae (*Diversispora* VTX00062, VTX00354) and 2 (OTU012 and OTU019) to Paraglomeraceae (*Paraglomus* VTX00335, VTX00351). Although about only one third of the 31 OTUs were common to the three sites, this third included 69.5% of the retrieved OTU units, and five genus/clades out of 8, with only *Funnelliformis/Septoglomus*, *Acaulospora* and *Scutellospora* being excluded (Fig 3 and S2 Table).

Two (OTU018, OTU031), three (OTU014, OTU024, OTU028) and six AM fungal OTUs (OTU016, OTU021, OTU022, OTU023, OTU026, OTU030) were retrieved exclusively from the VAL, CLM and MIL sites, respectively. OTU010 belonging to the *Acaulospora* genus (VTX00023) was found in MIL and CLM. In the same two sites, were found also OTU007, which is affiliated to *Glomus* sensu lato (VTX00143) and OTU020, which belongs to *Rhizophagus*/*Sclerocystis*, VTX00204. On the other hand, OTU025, which belongs to *Claroideoglomus*, (VTX00193) was the only OTU that was shared by VAL and CLM. Finally, another 4 OTUs (OTU011, OTU015, OTU027 and OTU029), which were assigned to *Glomus* sensu lato (VTX00153, VTX00149, VTX00418 and VTX00342) were retrieved from both VAL and MIL, which also shared both of the unique OTUs affiliated to *Funneliformis*/*Septoglomus* (VTX00064) and *Scutellospora* sp. (VTX00049), respectively, that is, OTU013 and OTU009 (Fig 3A and 3B). Nevertheless, the indicator species analysis only identified OTU010 (*Acaulospora* sp. VTX00023), which was mainly found in the MIL site (IndVal, 0.8 [*P*, 0.03]).

**Fig 4:**
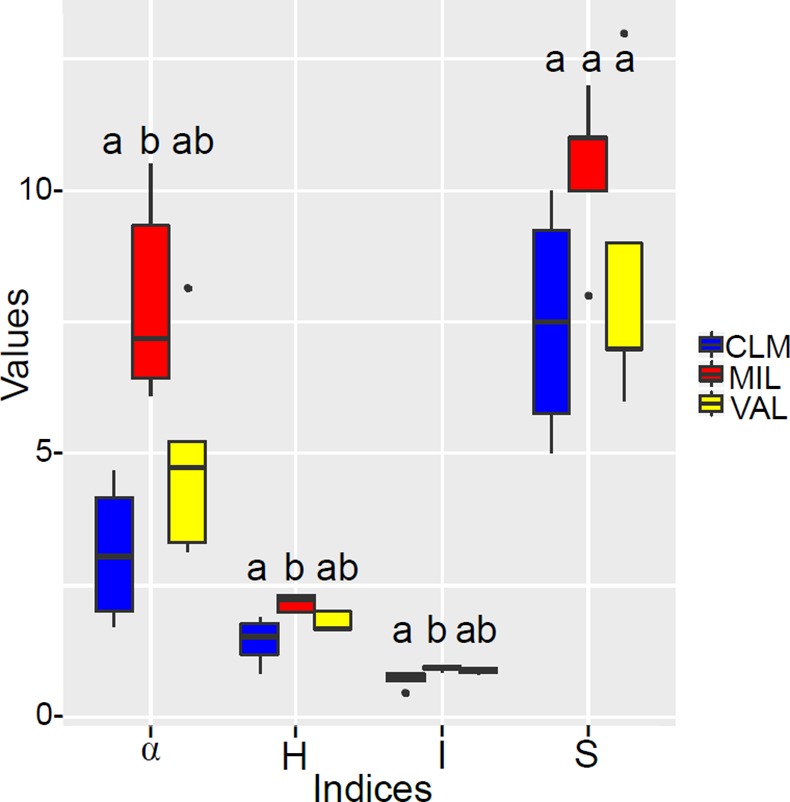
Box plot showing AMF diversity plots and the richness values of each site. The taxon richness (S), Shannon diversity (H), Simpson’s dominance (I), and Fisher’s alpha (alpha) values are reported. Boxplots with the same letter are not significantly different (p > 0.05), according to the non-parametric Nemenyi–Damico–Wolfe–Dunn post hoc test. CLM, Bassa di Colombart; MIL, Millefonts; VAL, Valcavera.

MIL showed a significantly higher diversity than CLM in all the diversity indices (α, H and I), while VAL was never significantly different from the other sites (Fig 4). Unlike the AMF diversity, the AMF richness was not significantly different in the three sites, although MIL harboured the higher species richness (Fig 4).

### Factors that shape the root colonization and AMF community composition

The percentage of total AMF colonization in the plots was positively correlated to the available extractable calcium and potassium (Ca and K), CEC, EC and FC, as shown in Table 3. The percentages of AMF arbuscules and vesicles were positively correlated to FC. Moreover, a positive correlation was found between the percentages of vesicles and DSE colonization (Table 3). Vegetation coverage was unrelated to AMF root colonization in *B*. *subacaulis* (Table 3).

**Table 3:** Correlation between the Percentage of AMF Root Colonization, Soil Properties, Soil Morphology, Vegetation Coverage and DSE Colonization Calculated Using the Kendal Tau.

Variables	Total	A	V
TN	0.278	0.144	-0.022
AC	-0.233	0.167	-0.112
TC	-0.376	-0.155	-0.211
Ca	**0.508**	0.243	0.211
K	**0.461**	0.281	0.362
Mg	0.124	-0.169	0.090
Na	0.196	0.012	0.139
C/N	-0.100	-0.233	-0.223
OM	0.258	0.101	-0.023
pH	-0.402	-0.324	-0.104
CEC	**0.442**	0.221	0.233
EC	**0.515**	0.328	0.153
FC	**0.530**	**0.442**	**0.544**
SL	-0.139	0.023	-0.105
BS	0.290	0.087	0.015
SC	-0.208	0.061	-0.234
VC	0.077	-0.026	0.193
DSE	0.244	0.333	**0.503**
MS	-0.011	0.149	0.207

A, arbuscular colonization; V, vesicular colonization; TN, total nitrogen; AC, active carbonate; TC, total carbonate; Ca, K, Mg, Na (available extractable nutrients); C/N, carbon/nitrogen ratio; OM; organic matter; CEC, cation exchange capacity; EC, electrical conductivity; FC, field capacity; SL, recorded slope; BS, bare soil; SC, stone coverage; VC, vegetation coverage; DSE, dark septate endophyte colonization; MS, microsclerotia colonization. Significant correlations are marked in bold (P < 0.05)

The ANOSIM analysis indicated a significant difference in the similarity of the vegetation relevés (R = 0.9692 p<0.001) between localities. In particular, VAL showed a significantly higher plant species richness than MIL, while CLM was never significantly different from the other sites (S3 Fig). Indicator species analysis identified from three to four species which were significantly associated to each site (S3 Table). No significant correlations were found between plant and fungal diversity indices.

**Fig 5:**
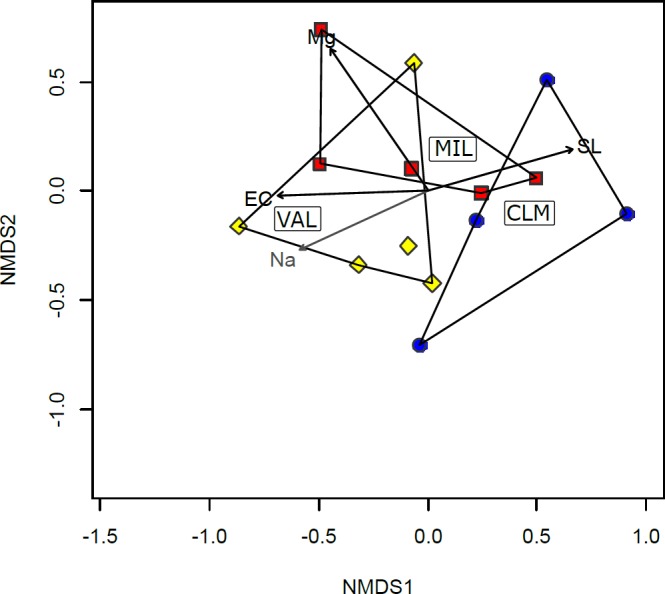
Joint plot of the NMDS ordination of the AMF communities colonizing the *B*. *subacaulis* roots in the different plots and the significance vectors (p < 0.1) of the environmental variables across the sites. The vectors graphically represent the correlations of the NMDS axes to each of the measured variables of the soil. The length of the arrow is proportional to the strength of the correlation between the environmental variables and community dissimilarities. Mg, available magnesium; Na, available sodium; EC, electrical conductivity; SL, slope. Polygons have been used to group the plots in the same site. Symbols: red, filled squares = MIL, Millefonts; blue,filled circles = CLM, Bassa di Colombart, and yellow, filled diamonds = VAL,Valcavera.

The NMDS ordination of the AMF community composition showed an acceptable stress level (0.19), thus indicating a good representation of the AMF taxa composition. The NMDS ordination did not show a clear separation of plots according to the sampling sites (Fig 5). Four out of seventeen variables of the soils (Na, Mg, EC and slope) fitted onto the NMDS as vectors, and showed a significant correlation (p < 0.1) with the AMF community composition (Fig 5). Among these variables, the available extractable magnesium (Mg), EC and slope were the variables most closely related to the AMF community composition in the plots (p < 0.05).

## Discussion

### Root colonization and AMF diversity in *B*. *subacaulis*

The AMF in the *B*. *subacaulis* roots showed a comparable degree of colonization (37–60%) with those recorded in other sites with disturbed soils [56]. The root colonization percentages largely exceeded those found by Binet et al. [20] in *Artemisia umbelliformis*, a plant that grows on alpine calcareous bedrocks in Switzerland, which are very poor in phosphorous nutrients, and confirmed that AMF root colonization is not depressed at altitudes of between approximately 2000 and 3000 m asl ([19]-[23]) in the Alps.

The AMF communities of the *B*. *subacaulis* roots were identified in this work for the first time on the basis of 18S amplicons. Amplification with the nested PCR approach gave a wide coverage of the Glomeromycota phylum, including three out of four orders and six out of the ten Glomeromycota families ([36],[43]). All the detected OTUs corresponded to already known virtual taxa, thus suggesting that, although the diversity of the Glomeromycota phylum had been overlooked in many ecosystems [57], the recent studies that have investigated AMF in continents, geographical regions and biomes ([58],[59]) have covered most of the AMF diversity.

Most of the 31 OTUs belonged to Glomerales (Glomeraceae 52% and Claroideoglomeraceae 22%), which are known to dominate AMF communities in many ecologically different environments ([39],[41],[60–63]). The remaining 26% of the OTUs was composed of Diversisporales (Diversisporaceae 10%, Acaulosporaceae 3% and Gigasporaceae 3%) and Paraglomerales (Paraglomeraceae 10%). The most represented family among the Glomerales was that of Glomeraceae, with *Glomus* sensu lato and *Rhizophagus*/*Sclerocystis* being very abundant in the root samples. The scree substrates in which *B*. *subacaulis* lives consist of mobile rocky debris of different sizes that can cause repeated fragmentations of the mycelial network, and they likely induce a selection of disturbance-tolerant AMF phylotypes, as already shown for other types of habitat [26]. Therefore, the abundance of *Glomus* sensu lato is not surprising. In fact, *Glomus* species have frequently been found in physically disturbed habitats, such as in agricultural landscapes ([56],[64],[65]), restored semi-natural grasslands [26] and coastal sand dunes [66]. The abundance of *Glomus* in disturbed habitats has been related to its high capacity to sporulate [64], to colonize roots from AM root fragments [67] and to readily form anastomoses [68]. All these characteristics may increase the competitive ability of these fungi and the rapidity and extent at which the external mycelia develop in soil.

### AMF community variations between the sampling sites

Roughly one third of the 31 OTUs retrieved from the roots of *Berardia* were found in the three sites. These OTUs were phylogenetically related to 5 genus/clades out of 8, and most of them were characterized by a high number of OTU units, retrieved from the roots (S2 Table). Thus, a core of AMF taxa presumably colonizes a large part of the *Berardia* roots. A similar situation, even though at a much larger scale, was found in potato roots in the Andes [69], where certain *Acaulospora*, *Cetraspora*, *Claroideoglomus* and *Rhizophagus* formed an AMF core-species community that had remained conserved over a wide range of environmental conditions. These results could indicate an important role of the plant species in structuring the host fungal community [24]. However, because some of the core-AMF of *B*. *subacaulis* were phylogenetically related to VTX00222, VTX00113, and VTX00193, which are abundant in other continents and climatic zones [59], our findings are also in agreement with the idea that some AMF taxa are distributed throughout the world. A shared pool of geographically widespread non-host-specific taxa, in fact, might be present in many different ecosystems, probably as a result of their efficient spore dispersal ([5],[70]). It was not the scope of this work to establish which of the two mechanisms could determine the *Berardia* AMF core-community.

One third of the OTUs were only retrieved from one site (2 in VAL, 3 in CLM and 6 in MIL). The other OTUs were found in two sites, with MIL and VAL sharing the highest number of OTUs. Nevertheless, an indicator species analysis, which accounts for both the abundance and frequency of species in sampling sites, identified only OTU010 (*Acaulospora* sp. VTX00023) as an indicator of a specific site (MIL). These results are in agreement with the significantly higher diversity recorded in MIL than in CLM (Fig 4). The significant association between MIL and *Acaulospora* deserves further investigation to establish the potential ecological functions of this AM genus. In fact, Acaulosporaceae sequences have frequently been detected in plant roots from very different highlands in Europe and in other continents [71], and species of the *Acaulospora* genus have been found associated with several pioneer plants of subnival and nival scree communities ([22],[69]).

The diversity and composition of AMF communities varied between the sites, as shown by the significantly higher values of α, H and I at the MIL site than at CLM (Fig 4). The level of richness of the AMF communities detected in the *Berardia* roots was in the same range as those previously detected in some plants at high altitudes in the Alps [72], and was generally lower than those found at lower altitudes [62]. Nevertheless, the diversity indices of colonization recorded at the site with the lowest altitude (MIL) were not significantly higher than those of the highest altitude’s site (VAL) while they were higher than those found at the intermediate altitude (CLM). This lack of an altitudinal trend suggests that altitude is not a determinant of the AMF community in *B*. *subacaulis*.

### Factors that affect root colonization and AMF communities

Both biotic and abiotic factors have been shown to influence the intensity of AM root colonization [5]. However, the AMF colonization in this study was not related to vegetation coverage or to DSE colonization, with only the vesicles being directly related to DSEs (Table 3). High DSE levels have typically been documented in alpine plants since the first studies that were conducted in mountain habitats [73], and they occur up to very high elevations [23]. Intraradical AM vesicles are lipid storage nutrient structures whose development is stimulated by less favourable conditions for root growth [74], which could instead favour root colonization by DSE. However, this possibility still needs to be confirmed, because the few studies reported so far on the interactions between these two common root symbionts have given conflicting results. Both competition and facilitation between AMF and DSEs have been documented ([75],[76] and references therein), thus pointing to a heterogeneous response that may depend on environment-plant species interactions.

When abiotic soil parameters are considered, it can be seen that the AMF colonization of *Berardia* roots is positively correlated to the available calcium and potassium (Ca and K), CEC, EC and FC (Table 3). Soil CEC is indicative of the capacity of soil to retain positively-charged ions, and it influences the soil structure and stability [77]. The latter is also influenced, at least in part, by the abundance of AMF. It is in fact known that: (1) the bulk of the AMF organisms are formed by the extraradical mycelium [74], and a relation exists between the length of the colonized roots and that of the extraradical hyphae [78]; (2) the structure of the soil is improved directly by the extraradical AM hyphae that extend from the host roots into the substrate, and enmesh soil particles and, indirectly, by their production of glomalin, a glycoprotein involved in the formation of water-stable soil aggregates ([79],[80]). The cation exchange capacity influences EC [81], and may be related to FC, because water retention and availability has been shown to be higher in well-structured and colonized soils [5].

The plant communities associated to *B*. *subacaulis* significantly differed between sites with regards to diversity, species richness and site-specific species. The latter are mainly mycorrhizal and, by hosting different AMF taxa and/or richness, the possibility exists that they could influence the AMF communities of *B*. *subacaulis* both directly and indirectly, through modifying the soil properties. However, this has been shown not to occur, in accords with the suggestion of [82] that plant species identity may be less important than other factors in structuring local AMF communities.

The NMDS ordination confirmed that the community composition of AMF changed from plot to plot, and was affected by soil parameters related to salinity (EC, Mg and Na) plus slope (Fig 5). This result is in line with previous findings that showed that soil salinity may influence the distribution of AMF in agricultural soils [83]. Moreover, even if to our knowledge, the impact of slope on AMF diversity has not been reported in the literature, it probably affects both water and ion leakage and the stability of the substrate. In fact, slope had the opposite effect on the AMF diversity of the soil salinity parameters (Fig 5). Moreover, because of the increasing downward movement of rock fragments and soil, slope may contribute to mechanical disturbance of the substrate in mountain screes, probably physically disrupting AM hyphal networks, as it occurs in ploughed soils, and this may affect the AMF community, as mentioned above.

## Conclusions

In the present study, we have investigated fungal diversity in an environment that has so far been studied little, but which is very common in the Alps, calcareous scree slopes. The plant species in this harsh environment are subjected to extreme climatic and edaphic variable ranges, and they have to adapt to debris falls and substrate movements.

Arbuscular mycorrhizal fungi are known for their capacity to improve plant performances in hostile environments and under different stresses, especially drought and poor soil quality.

This study has demonstrated that the abundance and diversity of AMF in *B*. *subacaulis* roots is related to the chemical and physical properties of the soil and, according to the literature, has suggested a role of AMF in improving soil quality. Moreover, it has shown that also the slope influences AMF diversity.

Although the soil profiles of Alpine screes differ very little, these screes are very heterogeneous, as far as the rate of the substrate movements in function of the slope and debris sizes is concerned. Despite the established relationships between the occurrence of AM fungi and the physical disturbance of soils in an agronomic context, to date few studies have examined the effects of slope on AM fungal diversity and abundance in a natural ecosystem. We suggested that the aforementioned heterogeneity of Alpine screes might have contributed to the selection of AMF taxa with different degrees of disturbance tolerance, thus creating the conditions for a variegated situation of plant-fungus assemblages.

The study of the interactions between threatened plants of extreme habitats, such as *B*. *subacaulis*, and the associated AMF is gaining importance because of their implications on species conservation, especially in view of the ongoing climate changes. In fact, variations in the occurrence and diversity of AMF can influence the stability and population dynamics of an ecosystem by changing plant competiveness and persistence.

## Supporting information

S1 Fig Capitulum and plant (in the box) of *Berardia subacaulis*. (PDF)

S2 Fig Species accumulation curve. (PDF)

S3 Fig Box plot showing plant species richness of each site. (PDF)

S1 Table Soil properties and morphological characteristics of *Berardia subacaulis* screes. (PDF)

S2 Table List of operational taxonomic units (OTUs) of the AMF sequences retrieved from *Berardia subacaulis* roots. (PDF)

S3 Table Results of the Indicator Species Analysis on the vegetation data. (PDF)
