# Supplementary figures and images for: The abundance and diversity of arbuscular mycorrhizal fungi are linked to the soil chemistry of screes and to slope in the Alpic paleo-endemic Berardia subacaulis
Source: PLoS One. 2017 Feb 13;12(2):e0171866. doi: 10.1371/journal.pone.0171866 (PMC5305098; doi:10.1371/journal.pone.0171866)

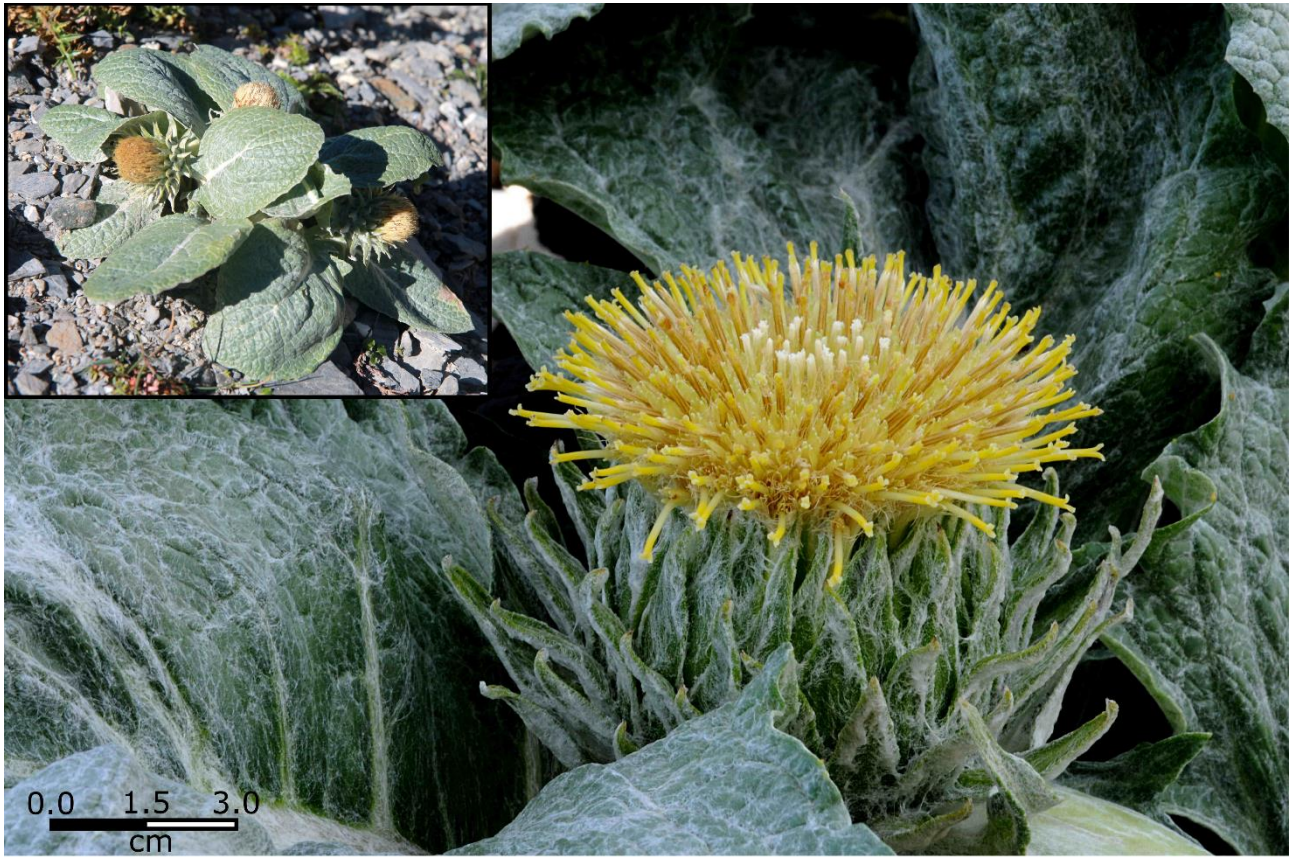

**S1 Fig. Capitulum and plant (in the box) of *Berardia subacaulis*.**

Supplement: S1 Fig — (PDF) [file pone.0171866.s001.pdf]
