# Supplementary material for: The abundance and diversity of arbuscular mycorrhizal fungi are linked to the soil chemistry of screes and to slope in the Alpic paleo-endemic Berardia subacaulis
Source: PLoS One. 2017 Feb 13;12(2):e0171866. doi: 10.1371/journal.pone.0171866 (PMC5305098; doi:10.1371/journal.pone.0171866)
Supplement: S2 Fig — (PDF) [file pone.0171866.s002.pdf]

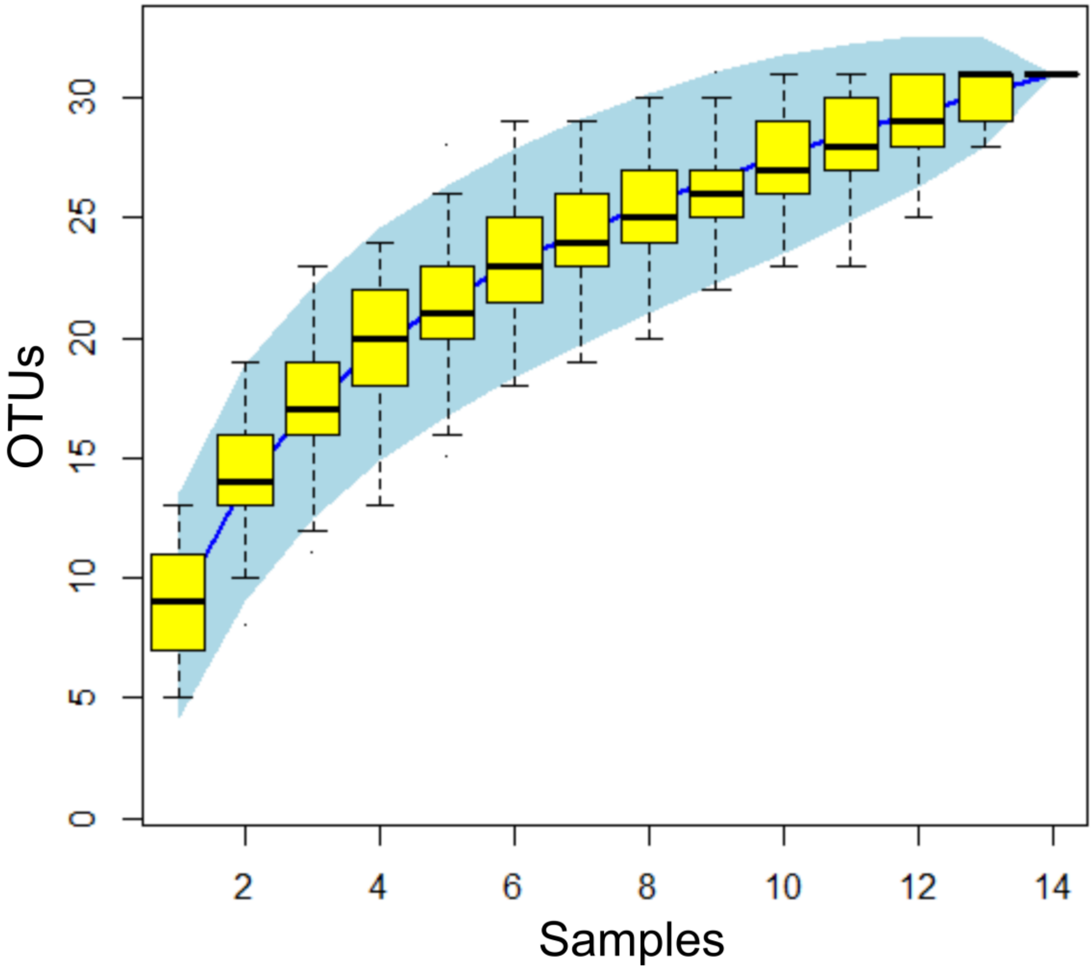


**S2 Fig. Species accumulation curve.** To examine whether the number of OTUs increases as the sample size increases.
