# Supplementary material for: The abundance and diversity of arbuscular mycorrhizal fungi are linked to the soil chemistry of screes and to slope in the Alpic paleo-endemic Berardia subacaulis
Source: PLoS One. 2017 Feb 13;12(2):e0171866. doi: 10.1371/journal.pone.0171866 (PMC5305098; doi:10.1371/journal.pone.0171866)
Supplement: S3 Fig — (PDF) [file pone.0171866.s003.pdf]

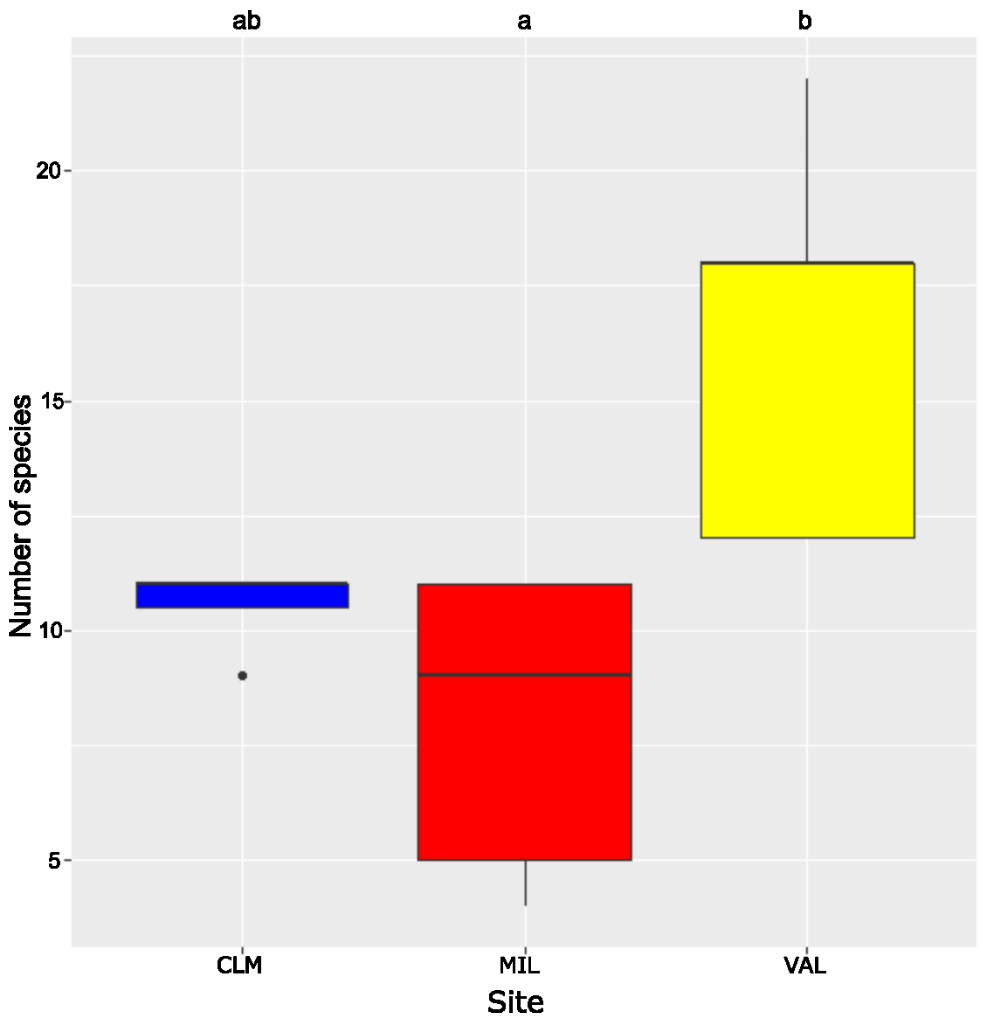

S3 Fig. Box plot showing plant species richness of each site. Boxplots with the same letter are not significantly different ($p > 0.05$), according to the non-parametric Nemenyi–Damico–Wolfe–Dunn post hoc test. Bassa di Colombart, CLM; Millefonts, MIL; Valcavera, VAL.
