# Supplementary material for: The abundance and diversity of arbuscular mycorrhizal fungi are linked to the soil chemistry of screes and to slope in the Alpic paleo-endemic Berardia subacaulis
Source: PLoS One. 2017 Feb 13;12(2):e0171866. doi: 10.1371/journal.pone.0171866 (PMC5305098; doi:10.1371/journal.pone.0171866)
Supplement: S1 Table — (PDF) [file pone.0171866.s004.pdf]

S1 Table. Soil properties and morphological characteristics of *Berardia subacaulis* screes. Soil data includes total nitrogen (TN), active carbonate (AC), total carbonate (TC), cation exchange capacity (CEC), electrical conductivity (EC), available extractable nutrients (Ca, K, Mg, Na, P), carbon/nitrogen ratio (C/N), organic matter (OM), pH, field capacity (FC). recorded slope (SL), stone coverage (SC) and bare soil (BS). Percentage values of vegetation coverage (VC) have also been indicated. CLM, Bassa di Colombart; MIL, Millefonts and VAL, Valcavera.

	CLM1	CLM2	CLM3	CLM4	MIL1	MIL2	MIL3	MIL4	MIL5	VAL1	VAL2	VAL3	VAL4	VAL5
TN (g/kg)	1.7	1.1	1	1.1	1.3	1.8	1.6	0.8	0.6	4	4.9	2.9	4.1	3.3
AC (g/kg)	75	50	48	51	28	47	35	25	23	29	0	28	18	43
TC (g/kg)	614.8	426	416.9	540.1	540	360.5	467.7	606	681.8	349.8	11.9	294	96.6	423
Ca (meq/100g)	6.64	7.18	7.15	7.03	9.18	7.86	9.43	5.68	5.9	14.43	21	11.33	17.27	11.25
K (meq/100g)	0.2	0.23	0.26	0.19	0.23	0.24	0.25	0.17	0.17	0.28	0.35	0.24	0.3	0.18
Mg (meq/100g)	0.12	0.09	0.12	0.08	0.62	0.14	0.25	0.2	0.31	0.16	0.28	0.11	0.28	0.11
Na (meq/100g)	0.18	0.19	0.15	0.16	0.18	0.22	0.21	0.18	0.2	0.3	0.24	0.23	0.2	0.18
P (mg/kg)	ND	ND	ND	ND	ND	ND	7.00	3.00	ND	2.00	ND	2.00	ND	ND
C/N	5.9	5.5	5	4.5	8.5	9.4	9.4	8.8	8.3	7.3	8	6.9	7.8	7
OM (g/kg)	17.2	10.3	8.6	8.6	19	29.3	25.9	12.1	8.6	50	67.2	34.5	55.2	39.7
pH	8.5	8.4	8.5	8.4	8.5	8.4	8.4	8.5	8.5	8.3	8	8.4	8.2	8.4
CEC (meq/100g)	8.7	8.6	9.7	7	12.3	8.5	11.3	6.8	8.3	13	23.8	13.6	20.8	14.7
EC (dS/m)	0.09	0.09	0.08	0.09	0.12	0.11	0.12	0.07	0.08	0.15	0.14	0.12	0.13	0.12
FC	20.6	29.5	32.7	21.3	28.7	18.2	24.5	19.4	18.5	21.9	33	22.2	30.3	24.8
SL (°)	30	31.67	36.67	38.33	30	36.67	26.67	28.33	31.67	20	31.67	30	35	35
SC (%)	95	90	85	90	95	95	80	90	85	90	85	80	60	90
BS (%)	0	0	0	5	0	0	0	5	0	5	5	5	20	0
VC (%)	5	10	15	5	5	5	20	5	15	5	10	10	20	10

ND, not detectable.
