# Supplementary material for: The abundance and diversity of arbuscular mycorrhizal fungi are linked to the soil chemistry of screes and to slope in the Alpic paleo-endemic Berardia subacaulis
Source: PLoS One. 2017 Feb 13;12(2):e0171866. doi: 10.1371/journal.pone.0171866 (PMC5305098; doi:10.1371/journal.pone.0171866)
Supplement: S2 Table — (PDF) [file pone.0171866.s005.pdf]

S2 Table. List of the operational taxonomic units (OTUs) of the AMF sequences retrieved from *Berardia subacaulis* roots. Taxonomic affiliation, OTU correspondence with the closest virtual taxon (VT) after blast search against the MaarjAM database (Öpik et al. 2010), first hit sequence code, Pairwise % identity (P%I), alignment length and corresponding accession number are also reported. CLM, Bassa di Colombart; MIL, Millefonts and VAL, Valcavera.

OTU_ID	CLM				MIL					VAL					Total OTU units	Order	Family	Genus/Clade	Virtual Taxon	first hit sequence code	P%I	Length	GenBank Accession number
	1	2	3	4	1	2	3	4	5	1	2	3	4	5									
OTU_001	5	12	2	2	0	3	0	3	3	0	1	4	2	3	*40*	Diversisporales	Diversisporaceae	Diversispora	VTX00062	Y17644	99.88	801	KY416573
OTU_002	1	0	1	1	0	0	3	1	2	1	0	4	5	0	*19*	Glomerales	Claroideoglomeraceae	Claroideoglomus	VTX00056	HE576876	99.88	806	KY416574
OTU_003	1	3	1	4	0	4	2	2	2	2	1	0	0	0	*22*	Glomerales	Claroideoglomeraceae	Claroideoglomus	VTX00193	HE614988	100.0	805	KY416575
OTU_004	15	0	0	10	0	0	9	0	4	3	5	13	0	7	*66*	Glomerales	Glomeraceae	Rhizophagus/Sclerocystis	VTX00113	JQ811204	100.0	795	KY416576
OTU_005	0	0	27	0	2	0	1	0	1	2	0	0	0	0	*33*	Glomerales	Glomeraceae	Glomus *sensu lato*	VTX00342	FR693450	98.87	794	KY416577
OTU_006	1	0	0	0	2	3	0	2	0	0	1	1	1	0	*11*	Diversisporales	Diversisporaceae	Diversispora	VTX00354	HE615058	97.63	801	KY416578
OTU_007	0	11	2	3	0	3	0	1	0	0	0	0	0	0	*20*	Glomerales	Glomeraceae	Glomus *sensu lato*	VTX00143	DQ085180	99.12	794	KY416579
OTU_008	0	0	1	9	1	5	1	9	2	0	0	1	2	3	*34*	Glomerales	Glomeraceae	Glomus *sensu lato*	VTX00222	GU059535	99.69	647	KY416580
OTU_009	0	0	0	0	1	0	2	0	0	0	5	1	2	0	*11*	Diversisporales	Gigasporaceae	Scutellospora	VTX00049	FJ009672	99.12	793	KY416581
OTU_010	0	1	0	1	3	2	0	2	1	0	0	0	0	0	*10*	Diversisporales	Acaulosporaceae	Acaulospora	VTX00023	JF414186	98.25	801	KY416582
OTU_011	0	0	0	0	0	0	1	0	0	0	0	1	5	0	*7*	Glomerales	Glomeraceae	Glomus *sensu lato*	VTX00153	DQ085239	99.12	798	KY416583
OTU_012	0	0	0	2	0	4	0	2	0	0	1	1	0	1	*11*	Paraglomerales	Paraglomeraceae	Paraglomus	VTX00335	HE576839	100.0	799	KY416584
OTU_013	0	0	0	0	0	2	0	1	0	4	0	1	0	9	*17*	Glomerales	Glomeraceae	Funneliformis/Septoglomus	VTX00064	HE614970	99.7	669	KY416585
OTU_014	7	0	0	0	0	0	0	0	0	0	0	0	0	0	*7*	Glomerales	Glomeraceae	Rhizophagus/Sclerocystis	VTX00105	HE615045	99.12	795	KY416586
OTU_015	0	0	0	0	1	0	3	0	0	7	0	0	0	0	*11*	Glomerales	Glomeraceae	Glomus *sensu lato*	VTX00149	HE576925	98.99	794	KY416587
OTU_016	0	0	0	0	0	3	0	0	0	0	0	0	0	0	*3*	Paraglomerales	Paraglomeraceae	Paraglomus	VTX00335	HE615075	96.24	798	KY416588
OTU_017	0	0	0	2	1	0	1	4	2	0	0	1	0	1	*12*	Glomerales	Claroideoglomeraceae	Claroideoglomus	VTX00055	JN252441	99.75	805	KY416589
OTU_018	0	0	0	0	0	0	0	0	0	2	0	0	0	0	*2*	Glomerales	Glomeraceae	Glomus *sensu lato*	VTX00159	EU340309	96.87	798	KY416590
OTU_019	2	0	0	0	0	0	0	1	0	0	0	2	0	2	*7*	Paraglomerales	Paraglomeraceae	Paraglomus	VTX00351	HE576800	99.88	803	KY416591
OTU_020	1	0	0	0	1	0	0	0	0	0	0	0	0	0	*2*	Glomerales	Glomeraceae	Rhizophagus/Sclerocystis	VTX00204	EU417637	98.24	795	KY416592
OTU_021	0	0	0	0	0	0	0	0	1	0	0	0	0	0	*1*	Glomerales	Glomeraceae	Glomus *sensu lato*	VTX00153	FR693497	97.36	795	KY416593
OTU_022	0	0	0	0	0	0	0	0	1	0	0	0	0	0	*1*	Glomerales	Glomeraceae	Glomus *sensu lato*	VTX00418	FN869856	98.36	795	KY416594
OTU_023	0	0	0	0	0	1	0	0	0	0	0	0	0	0	*1*	Glomerales	Glomeraceae	Glomus *sensu lato*	VTX00342	HE615029	93.2	794	KY416595
OTU_024	1	0	0	0	0	0	0	0	0	0	0	0	0	0	*1*	Glomerales	Claroideoglomeraceae	Claroideoglomus	VTX00056	HE615008	96.76	802	KY416596
OTU_025	0	3	0	0	0	0	0	0	0	0	0	1	0	0	*4*	Glomerales	Claroideoglomeraceae	Claroideoglomus	VTX00193	HE615035	98.5	799	KY416597
OTU_026	0	0	0	0	0	1	0	0	0	0	0	0	0	0	*1*	Glomerales	Claroideoglomeraceae	Claroideoglomus	VTX00193	HE615035	96.62	799	KY416598
OTU_027	0	0	0	0	0	0	0	0	2	0	0	1	0	0	*3*	Glomerales	Glomeraceae	Glomus *sensu lato*	VTX00418	HE576874	97.23	795	KY416599
OTU_028	1	0	0	0	0	0	0	0	0	0	0	0	0	0	*1*	Diversisporales	Diversisporaceae	Diversispora	VTX00062	Y17644	95.74	798	KY416600
OTU_029	0	0	0	0	0	0	0	3	0	2	2	0	0	0	*7*	Glomerales	Glomeraceae	Glomus *sensu lato*	VTX00342	HE615029	97.98	794	KY416601
OTU_030	0	0	0	0	0	0	1	0	0	0	0	0	0	0	*1*	Glomerales	Claroideoglomeraceae	Claroideoglomus	VTX00193	HE615072	98.63	801	KY416602
OTU_031	0	0	0	0	0	0	0	0	0	1	0	0	0	0	*1*	Glomerales	Glomeraceae	Glomus *sensu lato*	VTX00407	HE576818	97.1	794	KY416603
