# Supplementary material for: The abundance and diversity of arbuscular mycorrhizal fungi are linked to the soil chemistry of screes and to slope in the Alpic paleo-endemic Berardia subacaulis
Source: PLoS One. 2017 Feb 13;12(2):e0171866. doi: 10.1371/journal.pone.0171866 (PMC5305098; doi:10.1371/journal.pone.0171866)
Supplement: S3 Table — (PDF) [file pone.0171866.s006.pdf]

S3 Table. Results of the Indicator Species Analysis on the vegetation data. For the species significantly associated to the sites (p-value<0.05), the value of the correlation (IV) and the statistical significance of the association (p-value) are also reported.

Species	Site	IV	p-value
Saxifraga aizoides	CLM	1.0000	0.0012
Campanula scheuchzeri	CLM	0.8318	0.0043
Saxifraga oppositifolia	CLM	0.6976	0.0091
Galium pseudohelveticum	CLM	0.5915	0.0251
Galium verum	MIL	1.0000	0.0010
Athamantha cretensis	MIL	0.8412	0.0005
Helictotrichon sempervirens	MIL	0.5693	0.0480
Senecio doronicum subsp. *doronicum*	VAL	0.8000	0.0112
Anthyllis vulneraria subsp. *alpestris*	VAL	0.8000	0.0114
Oreochlora seslerioides	VAL	0.6535	0.0152

CLM, Bassa di Colombart; MIL, Millefonts and VAL, Valcavera.
